# Overweight and obesity among youth in a city in Western Amazonia: a time trend analysis from 2006 to 2024 of the largest geographic capital of Brazil

**DOI:** 10.1017/S1368980025101778

**Published:** 2025-12-26

**Authors:** Rafael Martins da Costa, Edson dos Santos Farias, Marta Carolina Terto de Morais, Giovanna Eduarda da Silva, Geovane Biet de Sousa, Luis Gonzaga de Oliveira Gonçalves, Silvia Teixeira de Pinho

**Affiliations:** 1 Department of Physical Education, Health Center, Federal University of Rondônia, Porto Velho, Rondônia, Brazil; 2 Postgraduate Program in Psychology, Health Center, Federal University of Rondônia, Porto Velho, Rondônia, Brazil; 3 Postgraduate Program in Physical Education, Federal University of Pelotas, Pelotas, Rio Grande do Sul, Brazil; 4 School of Physical Education and Physiotherapy, Federal University of Pelotas, Pelotas, Rio Grande do Sul, Brazil

**Keywords:** Adolescent health, children health, paediatric obesity, epidemiological monitoring

## Abstract

**Objective:**

Overweight and obesity have become a global public health concern, with prevalence rising sharply in low- and middle-income countries. This study analysed temporal trends in overweight and obesity among schoolchildren in the largest capital city of Brazil, from 2006 to 2024.

**Design:**

Repeated cross-sectional.

**Setting:**

Schoolchildren aged 6 to 19 years from Porto Velho, Rondônia, Brazil.

**Participants:**

A total of 12 646 participants were evaluated. To assess the BMI *z*-score, standardised body mass and height measurements were used, stratified by sex (male and female) and age group (6–10 and 11–19 years). Temporal trends were assessed using joinpoint regression analysis.

**Results:**

Overall, 17·8 % of participants were classified with overweight, and 8·6 % were classified with obesity. The study also found that obesity prevalence was consistently higher among younger age groups compared to adolescents. The results revealed a significant increase in obesity prevalence among girls (annual percentage change (APC) = 5·81 %; 95 % CI = 1·03–10·81; *P*-value = 0·021) and children aged 6 to 10 years (APC = 5·20 %; 95 % CI = 1·17–9·39; *P*-value = 0·017), while no significant trends were observed for overweight or for male adolescents or adolescents aged 11 to 19 years.

**Conclusions:**

Our findings indicate rising obesity among girls and children aged 6–10 years and support the need for urgency. We recommend targeted action, including implementing mandatory quality physical education and school nutrition standards, enforcing restrictions on marketing to children and prioritising municipal policies that increase access to healthy foods.

The global prevalence of overweight and obesity has increased at an alarming rate, evolving into a pandemic with profound public health implications. According to the WHO, obesity rates nearly tripled between 1975 and 2016, contributing to 3·4 million deaths and 4 % of global disability-adjusted life years in 2010^(1–3)^. Once concentrated in high-income countries, excess weight is expanding rapidly across low- and middle-income settings as urbanisation, economic transition and proliferating obesogenic environments reshape population risk, underscoring the urgent need for robust monitoring and effective interventions as the burden of obesity increasingly eclipses that of underweight^(3,4)^. Of particular concern is the rising prevalence among children and adolescents, given the strong tracking of excess weight into adulthood and its links with elevated lifetime risk of CHD, type 2 diabetes, several cancers and adverse psychosocial outcomes, including depression and low self-esteem^(5–7)^. In Brazil, a nation characterised by continental dimensions and profound socio-economic heterogeneity, the national burden of paediatric obesity mirrors this global trajectory, yet the underlying dynamics are highly stratified by geography and equity. Understanding these localised trends is essential for developing interventions that address the specific socio-ecological determinants driving the epidemic^(4,8,9)^.

Brazil’s pronounced regional disparities further complicate efforts to address overweight and obesity. The Southern and Southeastern regions, with greater industrialisation and economic development, contrast starkly with the Northern and Northeastern regions, which face significant social inequalities and limited access to healthcare services^(4)^. These disparities are particularly evident in the North, where the population is characterised by cultural and demographic diversity, including Indigenous peoples, riverbank dwellers and migrants from other regions of Brazil^(10)^. The North faces an acute ‘double burden of malnutrition’, where the persistence of historical nutritional deficits, evidenced by documented stature issues, coexists with rapidly accelerating rates of excess weight^(11,12)^. While a systematic review with meta-analyses suggests lower crude obesity prevalence compared to the more affluent Southern Brazil^(13)^, the Northern Brazil records the lowest prevalence of adequate feeding practices nationwide^(14)^. This rapid transition from undernutrition to caloric excess, often driven by increased consumption of ultra-processed foods, signals a volatile and highly vulnerable population^(14)^.

Porto Velho, the capital of Rondônia, serves as a crucial economic and demographic sentinel site within the Western Brazilian Amazon. Its designation as the largest municipality in Brazil by land area exacerbates administrative and public health challenges, including complex logistics for reaching remote communities often accessible only by river. This dynamic urban centre is currently undergoing rapid, often chaotic, urbanisation, which alters local food environments and contributes to increased sedentary behaviour^(15)^. Furthermore, the city and surrounding areas contend with compounding environmental health pressures, such as mercury contamination from regional artisanal gold mining activities^(16)^. Existing local surveillance data, although cross-sectional and limited in scope (2013–2016), already showed alarming combined prevalences of 18·8 % for overweight and 8·3 % for obesity among schoolchildren^(17)^, thus confirming the high local risk and validating the urgent need for robust, long-term monitoring.

Despite the clear epidemiological urgency and the alarming local prevalence estimates, robust, longitudinal temporal trend analyses spanning extensive time periods are severely lacking for school-aged children (6–19 years) in the Western Amazon. Previous investigations have often been restricted to shorter time frames or have focused exclusively on younger cohorts^(1)^. Furthermore, effective public health strategies require high-resolution data to identify the most vulnerable subgroups. Obesity dynamics are known to vary significantly by age and sex, influenced by differential exposures to obesogenic environments and physiological milestones, such as pubertal onset in girls^(1)^. Thus, a surveillance of temporal trends is critically necessary to pinpoint which specific age and gender groups are driving the accelerating epidemic in this unique, high-risk socio-ecological setting, information that is vital for the development of targeted, equitable interventions.

Given these disparities, the present study aims to analyse temporal trends in the prevalence of overweight and obesity among schoolchildren in Porto Velho, Rondônia. By investigating these trends, this study seeks to provide critical insights into the health challenges faced by adolescents in this region and contribute to the development of effective public health strategies to combat the obesity epidemic in Northern Brazil.

## Material and methods

### Study design and participants

This study used a repeated cross-sectional design, conducted in the Western Brazilian Amazon, specifically in Porto Velho, the capital of Rondônia, Brazil. Porto Velho has an estimated population of approximately 511 210 inhabitants and a Human Development Index (HDI) of 0·736. The dataset was provided by the Statistics Sector of the State Secretariat of Education (SEDUC) of Rondônia, covering the periods from 2006 to 2019 and 2022 to 2024. Data for 2020 and 2021 were unavailable due to disruptions caused by the COVID-19 pandemic.

Data collection was carried out through a partnership between SEDUC and the Center for Study and Research in Public Health (CEPESCO) at the Federal University of Rondônia. This collaboration involved projects under the Institutional Program for Scientific Initiation Scholarships (PIBIC), the Education Program for Health Work (PET-Saúde) and the Pedagogical Residency Program (PRP), as well as voluntary contributions from students in physical education, nursing and medicine programmes, alongside physical education teachers from the state school network. The data collected were systematically forwarded to SEDUC and stored in the statistics sector during the period from 2006 to 2024, excluding the COVID-19 pandemic years of 2020 and 2021.

Annual data were derived from SEDUC administrative records of school health assessments. Although some individual students could appear in more than one calendar year within the database, the dataset did not include consistent unique identifiers enabling deterministic longitudinal linkage at the individual level; therefore, the analyses treat each year’s sample as independent cross-sectional observations rather than a cohort followed over time. The inclusion criteria comprised all students enrolled and regularly attending school during the specified periods, with complete records of sex, age, body mass and height in the SEDUC statistical database. Exclusion criteria included incomplete participation across research stages, missing anthropometric data such as body mass or height, or the presence of physical deformities.

### Variables

#### Dependent variables

##### Overweight and obesity

Body mass was measured once using a calibrated scale with a precision of 0·1 kg. Stature was assessed using a portable stadiometer with a level of accuracy of 0·1 cm. These anthropometric measures were systematically performed and collected in the school environment by a multidisciplinary team composed of physical education teachers and students from health and education programmes as part of annual school health assessments. To ensure maximum standardisation and mitigate interobserver variability across the sixteen time points, a rigorous protocol was implemented annually, requiring ongoing training of new and existing team members (data not shown). All measurements followed standardised procedures with calibrated equipment, specifically a scale with 0·1 kg accuracy for body mass and a portable stadiometer with 0·1 cm accuracy for height, ensuring data reliability and comparability.

Weight status was determined based on BMI (kg/m^2^), adjusted for sex and age according to the WHO cut-off points. The calculated BMI values were converted into *z*-scores using the reference data provided by the WHO^(18)^: with overweight (≥*z*-score +1 < *z* + 2) and with obesity (≥ *z*-score +2). The software used to calculate overweight and obesity according to sex and age was WHO AnthroPlus.

### Independent variables

#### Years, sex and age group

The year of data collection was defined as the independent variable. The variable ‘year’ refers to the calendar (natural) year in which the anthropometric measurement was performed and recorded by SEDUC. Data corresponding to the calendar years 2020 and 2021 were not available due to the suspension of routine school health assessments during the COVID-19 pandemic and were therefore excluded from the analyses. Age (in completed years) was calculated from the difference between each participant’s date of birth and the measurement date. Participants were classified into two school groups that reflect the structure of Brazilian basic education: children (6–10 years old), corresponding to students typically enrolled in elementary school (1st to 5th grade) and adolescents (11–19 years old), corresponding to students typically enrolled in elementary school and high school (6th grade to 3rd grade; allowing for age heterogeneity due to grade repetition or delayed entry). This operational classification accommodates age heterogeneity arising from grade repetition or delayed school entry and guarantees that analyses are aligned with the administrative school framework used by the SEDUC. Stratified analyses were also conducted based on sex (male and female).

### Statistical analysis

Descriptive statistics were used to characterise the sample, with absolute and relative frequencies calculated. Temporal trends in overweight and obesity among adolescents were analysed using joinpoint regression analysis, a robust method for identifying changes in trend patterns over time. This method uses segmented linear regression with a logarithmic transformation and applies the Monte Carlo permutation test to determine if adding one or more joinpoints significantly improves the model fit over a single linear segment. Essentially, this technique determines whether multiple linear segments better represent the temporal trend than a single straight line, thereby enabling a more nuanced analysis.

The annual percentage change (APC) was calculated for each segment, accompanied by a 95 % CI. A positive APC indicates an increasing trend, while a negative APC signifies a decreasing trend. To provide a comprehensive summary of the temporal changes, the average annual percentage change (AAPC) was computed at the conclusion of the analysis period. For models with multiple joinpoints, the AAPC represents the weighted average of the APC across all identified segments; for single-segment models, the AAPC is equivalent to the APC.

Each joinpoint added to the model signifies a significant change in the linear trend, indicating whether a single straight line or additional segments provide a better representation of the data. The model was adjusted to allow for up to two joinpoints (a maximum of three segments) across the study period. A 5 % significance level was adopted to test the null hypothesis that both APC and AAPC were equal to zero. Trends were considered statistically significant when *P*-values were below 0·05 or when the 95 % CI was entirely positive (indicating an increasing trend) or entirely negative (indicating a decreasing trend).

Annual data on disease prevalence and outcomes were organised in Excel spreadsheets and subsequently imported into the Joinpoint Regression software (version 5.2.0) for analysis. This methodological framework ensured precise estimation of temporal changes, providing critical insights into the evolving prevalence of overweight and obesity among adolescents over the study period.

## Results

The sample consisted of 12 646 youth from Porto Velho, Rondônia, Brazil. The majority of participants were female (55·2 %) and aged between 11 and 19 years (71·5 %). Overall, 17·8 % of the participants were schoolchildren with overweight (55·6 % female and 76·9 % aged 11–19 years), while 8·6 % were schoolchildren with obesity (54·5 % male and 70·4 % aged 11–19 years). Detailed information for each year is presented in Table 1.

**Table 1.** Description of the prevalence of overweight and obesity, according to sex and age group, in young people from Porto Velho, Brazil, 2006–2024

Years					Sex								Age group							
	Total (*n* 12 646)				Male (*n* 5667)				Female (*n* 6979)				6–10 years old (*n* 3601)				11–19 years old (*n* 9045)			
	Overweight (*n* 2244)		Obesity (*n* 1093)		Overweight (*n* 997)		Obesity (*n* 596)		Overweight (*n* 1247)		Obesity (*n* 497)		Overweight (*n* 519)		Obesity (*n* 324)		Overweight (*n* 1725)		Obesity (*n* 769)	
	*n*	%	*n*	%	*n*	%	*n*	%	*n*	%	*n*	%	*n*	%	*n*	%	*n*	%	*n*	%
2006 (*n* 440)	98	4·4	52	4·8	52	5·2	39	6·6	46	3·7	13	2·6	3	0·6	2	0·6	95	5·5	50	6·5
2007 (*n* 980)	112	5·0	46	4·2	56	5·6	32	5·4	56	4·5	14	2·8	92	17·7	46	14·2	20	1·2	0	0·0
2008 (*n* 476)	54	2·4	20	1·8	20	2·0	16	2·7	34	2·7	4	0·8	46	8·9	16	4·9	8	0·5	4	0·5
2009 (*n* 378)	53	2·4	28	2·6	24	2·4	17	2·9	29	2·3	11	2·2	42	8·1	24	7·4	11	0·6	4	0·5
2010 (*n* 1301)	248	11·1	148	13·5	137	13·7	102	17·1	111	8·9	46	9·3	79	15·2	30	9·3	169	9·8	118	15·3
2011 (*n* 654)	125	5·6	22	2·0	64	6·4	16	2·7	61	4·9	6	1·2	29	5·6	9	2·8	96	5·6	13	1·7
2012 (*n* 1572)	332	14·8	164	15·0	128	12·8	75	12·6	204	16·4	89	17·9	0	0·0	0	0·0	332	19·3	164	21·3
2013 (*n* 2694)	461	20·5	165	15·1	216	21·7	83	13·9	245	19·7	82	16·5	0	0·0	0	0·0	461	26·7	165	21·5
2014 (*n* 819)	150	6·7	65	6·0	80	8·0	40	6·7	70	5·6	25	5·0	14	2·7	8	2·5	136	7·9	57	7·4
2015 (*n* 214)	42	1·9	35	3·2	19	1·9	24	4·0	23	1·8	11	2·2	9	1·7	12	3·7	33	1·9	23	3·0
2016 (*n* 800)	151	6·7	122	11·2	72	7·2	72	12·1	79	6·3	50	10·1	151	29·1	122	37·7	0	0·0	0	0·0
2017 (*n* 926)	181	8·1	85	7·8	0	0·0	0	0·0	181	14·5	85	17·1	11	2·1	6	1·9	170	9·9	79	10·3
2019 (*n* 315)	58	2·6	19	1·7	36	3·6	17	2·9	22	1·8	2	0·4	0	0·0	0	0·0	58	3·4	19	2·5
2022 (*n* 243)	37	1·7	24	2·2	17	1·7	12	2·0	20	1·6	12	2·4	0	0·0	0	0·0	37	2·1	24	3·1
2023 (*n* 154)	34	1·5	16	1·5	20	2·0	11	1·9	14	1·1	5	1·0	5	1·0	3	0·9	29	1·7	13	1·7
2024 (*n* 680)	108	4·8	82	7·5	56	5·6	40	6·7	52	4·2	42	8·5	38	7·3	46	14·2	70	4·1	36	4·7

*n*, absolute frequency; %, relative frequency in relation to the column.

**Figure 1.**
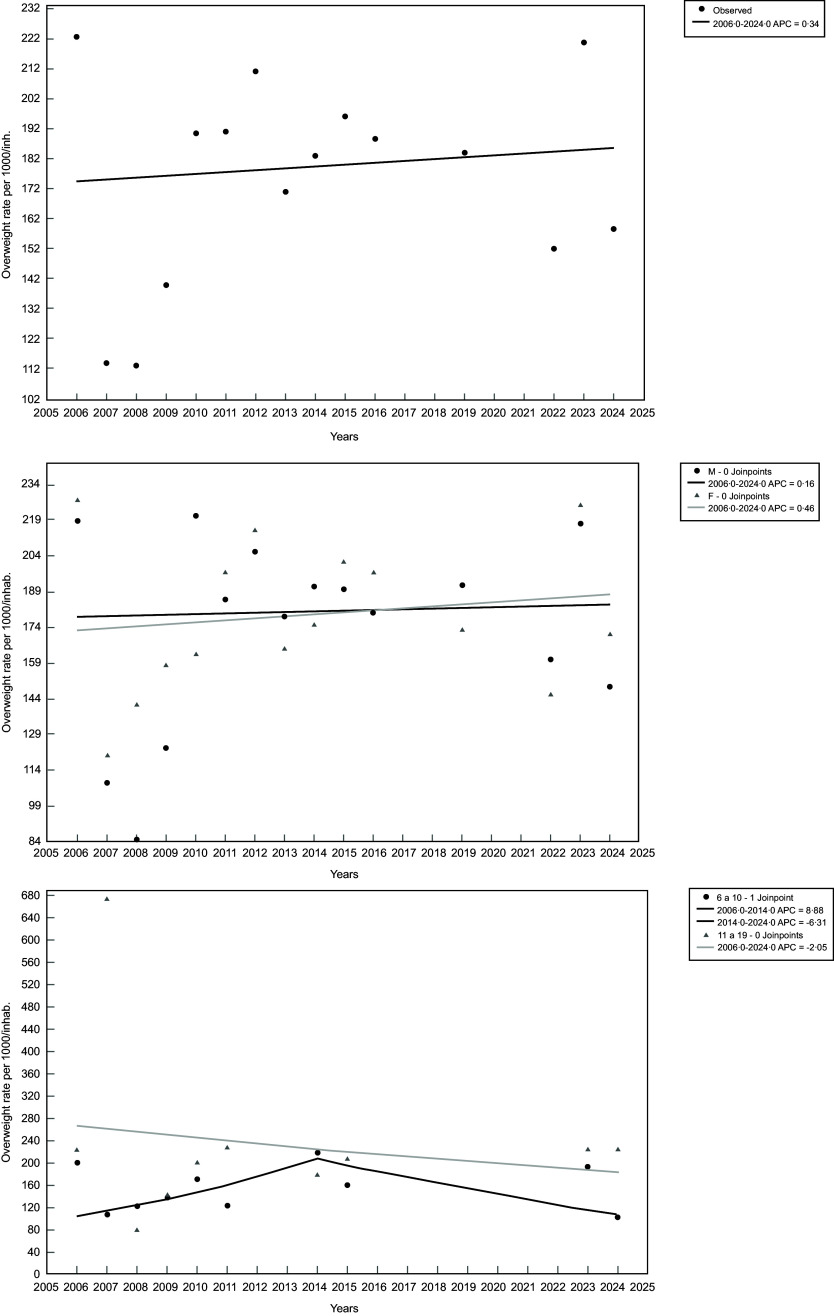
Overweight coefficients per 1000 inhabitants among youth from Porto Velho, Rondônia, Brazil, 2006–2024 (*n* 2244). Top section – overall results; middle section – results stratified by sex (M = male; F = female); bottom section – results stratified by age group (6–10 = 6 to 10 years old; 11–19 = 11 to 19 years old).

Regarding the temporal trend analyses, two joinpoints were identified (2006–2014 and 2014–2024) in the age group stratification. However, no significant temporal trends were observed for overweight among youth in Porto Velho, Brazil, either overall or when stratified by sex and age group (Table 2 and Figure 1).

**Table 2.** APC in overweight among young people in Porto Velho, Brazil, from 2006 to 2024 (*n* 2244)

Variables	Period	APC	95 % CI	*P*-value	AAPC	95 % CI	*P*-value	Trend
General	2006–2024	0·35	−1·99; 2·73	0·757	–		–	Ascending
Sex								
Male	2006–2024	0·16	−2·62; 3·02	0·905	–		–	Stationary
Female	2006–2024	0·46	−1·81; 2·78	0·672	–		–	Ascending
Age group								
6–10 years old	2006–2014	8·88	−3·73; 23·15	0·136	–		–	Ascending
	2014–2024	−6·31	−17·24; 6·05	0·234	–		–	Descending
	2006–2024	–		–	0·16	−6·34; 7·11	0·963	Stationary
11–19 years old	2006–2024	−2·05	−7·71; 3·95	0·444	–		–	Descending

APC, annual percentage change; AAPC, average annual percentage change.

**Figure 2.**
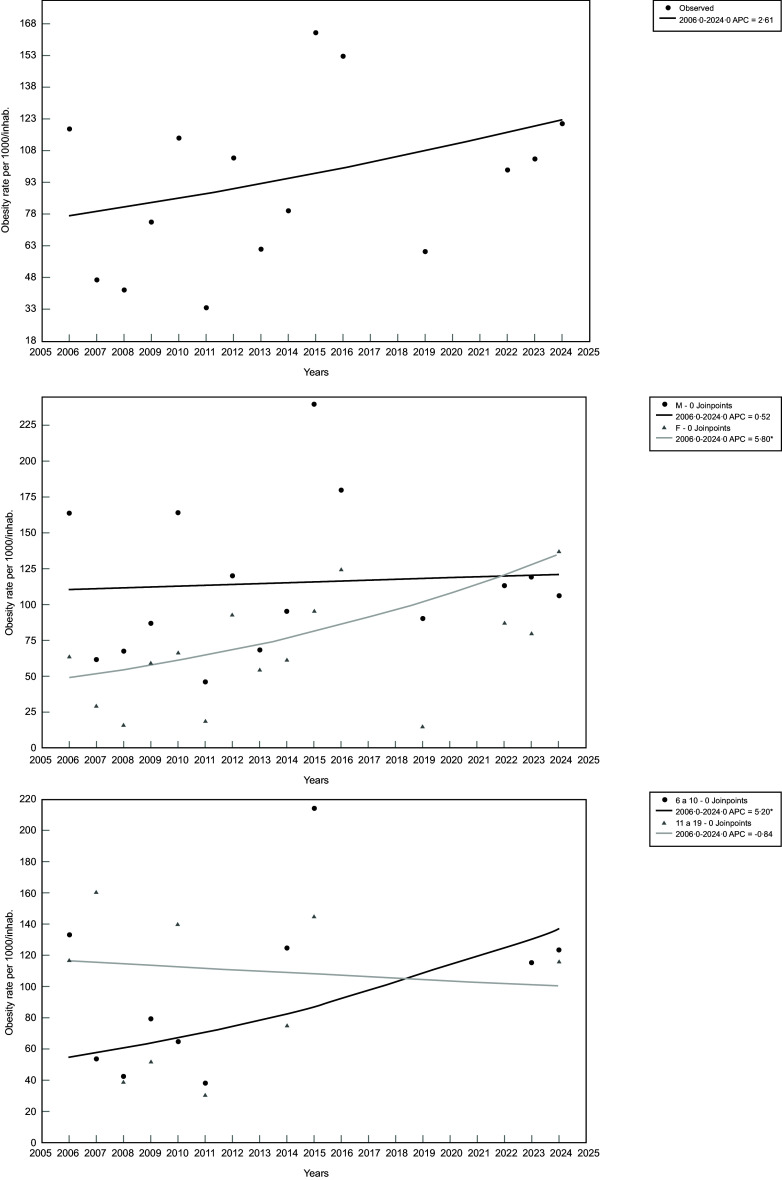
Obesity coefficients per 1000 inhabitants among youth from Porto Velho, Rondônia, Brazil, 2006–2024 (*n* 1093). Top section – overall results; middle section – results stratified by sex (M = male; F = female); bottom section – results stratified by age group (6–10 = 6 to 10 years old; 11–19 = 11 to 19 years old); *P-value <0.05.

For obesity, no joinpoints were identified in either the overall results or those stratified by sex and age group. When stratified by sex, we observed that female participants experienced an average annual increase of 5·81 % in obesity coefficients from 2006 to 2024 (Table 3 and Figure 2). No significant temporal trends were found among male participants. When stratified by age group, participants aged 6–10 years exhibited an average annual increase of 5·20 % in obesity coefficients during the same period (Table 3 and Figure 2). Similar to male participants, those aged 11–19 years did not show significant temporal trends in obesity coefficients.

**Table 3.** APC in obesity among young people in Porto Velho, Brazil, from 2006 to 2024 (*n* 1093)

Variables	Period	APC	95 % CI	*P*-value	Trend
General	2006–2024	2·61	−2·10; 7·55	0·258	Ascending
Sex					
Male	2006–2024	0·52	−4·64; 5·96	0·834	Stationary
Female	2006–2024	5·81	1·03; 10·81	0·021	Ascending
Age group					
6–10 years old	2006–2024	5·20	1·17; 9·39	0·017	Ascending
11–19 years old	2006–2024	−0·84	−6·20; 4·83	0·737	Descending

APC, annual percentage change.

## Discussion

The purpose of this study was to examine the temporal trends in overweight and obesity prevalence among schoolchildren of the largest capital of Brazil, Porto Velho, Rondônia. The present findings must be interpreted within the specific socio-ecological context of Porto Velho and the state of Rondônia. Porto Velho has experienced rapid and largely unplanned urban expansion, shifts in local food systems towards greater availability of industrially processed and ultra-processed products, and persistent deficits in basic infrastructure and health service access that disproportionately affect children in peri-urban and riverine communities^(19,20)^. Local surveillance and cross-sectional studies from Porto Velho and the Northern region document already-elevated prevalences of overweight and obesity in schoolchildren and highlight socio-economic and environmental vulnerabilities in these populations^(14,17)^. Together, these regional dynamics, that is, the accelerating nutrition transition (including greater intake of ultra-processed foods), constrained access to adequate sanitation and health services in parts of the municipality, school environment factors that limit physical activity and environmental exposures common to the Western Amazon, plausibly contribute to the accelerating obesity trend observed particularly among girls and children aged 6–10 years in our sample.

The lack of significant temporal trends in overweight prevalence may stem from Brazil’s unique socio-nutritional dynamics. Rapid industrialisation has driven shifts in dietary patterns, with a preference for energy-dense ultra-processed foods over minimally processed options, especially among urban populations^(1)^. At the same time, historical issues of undernutrition persist in less affluent regions, contributing to the coexistence of underweight and obesity within the same population^(4)^. This dual burden, coupled with socio-economic inequalities, could obscure clear trends in overweight rates. Additionally, the stabilisation of overweight prevalence might be influenced by the methodological differences among studies, such as variations in sample sizes or measurement accuracy^(21)^. These findings emphasise the urgent need for public health strategies to address both the undernutrition and obesity epidemics, particularly in regions undergoing rapid nutritional and economic transitions.

The lack of a consistent temporal trend in overall obesity prevalence may reflect the complex interplay of biological, behavioural and environmental factors influencing weight gain. Theories explaining the rapid rise in obesity over the past decades often cite increases in caloric intake, shifts in dietary composition, reduced physical activity and changes in the gut microbiome^(2)^. In Brazil, dietary transitions have been marked by increased consumption of ultra-processed foods, larger portion sizes and a shift towards meals consumed outside the home, particularly among adolescents^(5,22)^. These foods, characterised by high energy density, excessive sugar and unhealthy fats, are among the top ten consumed by Brazilian adolescents, exacerbating the obesity epidemic. However, the plateau in obesity trends observed in certain populations may indicate that these behavioural shifts are reaching a saturation point or that recent public health interventions are beginning to stabilise prevalence rates.

Another factor contributing to the absence of a clear temporal trend may be the limited reach and effectiveness of obesity-related policies and interventions. Systematic reviews^(23,24)^ highlight a lack of cohesive implementation and evaluation of policies targeting the food environment and behaviours in children and adolescents, particularly in middle-income countries^(23)^. While some nations have introduced taxes and regulations to reduce the consumption of energy-dense foods, fewer efforts focus on increasing access to affordable, healthy options such as fresh fruits and vegetables^(25)^. Additionally, socio-economic disparities in access to nutritious foods and healthcare interventions likely dilute the impact of these policies^(23)^. The stabilisation of obesity rates in some regions may also result from increased public awareness and modest behavioural changes, but sustained and equitable policy efforts are essential to achieve meaningful reductions in obesity prevalence across diverse populations^(23)^.

The observed gender-specific trend in obesity prevalence, with a significant increase identified only among girls, highlights the complex interplay of biological, behavioural and sociocultural factors influencing weight gain. Biological timing likely plays an important role. The hormonal changes that accompany puberty accelerate fat mass accrual in girls and thereby raise short-term risk of excess adiposity, which may predispose them to greater obesity risk compared to boys^(26–28)^. This mechanism is reinforced by local evidence from the Brazilian Amazon showing that early menarche is strongly associated with overweight and excess body fat: girls with early menarche presented a 35·5 % prevalence of excess weight and a 48·9 % prevalence of elevated body fat, substantially higher than peers with normal or late menarche^(26)^. Behavioural and social determinants further amplify this biological susceptibility. In the study region, girls tend to report higher levels of sedentary behaviour and lower participation in vigorous physical activity than boys, which reduces energy expenditure and facilitates weight gain over time^(17)^. Concurrently, the ongoing nutrition transition in Rondônia, that is, increasing availability and consumption of energy-dense, ultra-processed foods in homes and school environments, likely exposes girls to dietary risks that interact with both biological vulnerability and gendered patterns of activity^(14)^. Finally, while borderland dynamics may shape local markets and mobility, the preponderance of evidence indicates that rapid, often unplanned urbanisation, socio-economic disadvantage, and unequal infrastructure and service provision in Rondônia better explain the female-specific rise in obesity than a discrete cross-border effect^(14,29)^. Collectively, these biological, behavioural and contextual pathways offer a coherent rationale for the increasing trend observed among girls and underscore the need for sex-sensitive, school-based prevention strategies tailored to the Rondônia setting.

Sociocultural and environmental influences also play critical roles. Societal expectations surrounding body image may lead to dietary patterns and physical activity behaviours that predispose girls to obesity. Moreover, the urbanisation process in Brazil, characterised by increased consumption of ultra-processed, calorie-dense foods and reduced opportunities for physical activity, exacerbates these risks^(30,31)^. These dynamics, coupled with evidence suggesting a trend of earlier pubertal onset linked to excess weight, highlight the complex interrelation between nutritional, behavioural and hormonal factors in adolescent girls^(26)^.

The differential trends could also reflect varying exposure to obesogenic environments between boys and girls. Urbanisation, characterised by increased sedentary behaviours and the proliferation of screen-based activities, disproportionately affects girls, who may engage in fewer outdoor activities compared to boys^(30–32)^. Moreover, the heightened consumption of calorie-dense snacks, sweets and sugary beverages may be more pronounced among girls, driven by social habits and marketing strategies targeting this demographic^(5,26)^. These findings emphasise the need for gender-sensitive public health strategies to address the unique determinants of obesity among adolescent girls, including targeted interventions to improve dietary habits, reduce sedentary time and promote active lifestyles^(32)^.

The observed temporal trend of increasing obesity prevalence among children aged 6 to 10 years, contrasted with the absence of such a trend among adolescents aged 11 to 19 years, underscores the critical role of age-specific factors in shaping obesity dynamics. The higher susceptibility of younger children to obesity may be influenced by earlier exposure to obesogenic environments, including changes in dietary habits and reduced physical activity, which are particularly prominent during the foundational years of primary education. Evidence suggests that younger children experience a more rapid accumulation of BMI during this period, potentially exacerbated by disruptions in routine, such as those caused by the COVID-19 pandemic, which limited physical activity, increased sedentary behaviour and reduced access to healthcare services, including obesity prevention and management programmes^(33)^. These disruptions may have disproportionately affected younger children, whose developmental trajectories are more sensitive to environmental changes. A recent long-term panel from Florianópolis (2002–2018/19)^(34)^ similarly documented a sustained rise in obesity prevalence concentrated among children and public school attendees, while overweight trends were heterogeneous across age-sex strata. This pattern accords with our results that both studies identify subgroup-specific increases in obesity, underscoring that regional differences in obesogenic exposures may shape which age and sex groups drive local epidemics.

In contrast, the stabilisation of obesity prevalence among adolescents may reflect differences in growth patterns and the physiological milestones associated with puberty. Adolescents often experience a peak in height velocity that moderates BMI increases, a phenomenon documented in various populations, including those in the USA and Asia^(7)^. Additionally, older age groups may benefit from greater autonomy in making health-related decisions, including engagement in physical activity or healthier dietary practices, compared to younger children who are more dependent on family and school environments. Socio-economic factors also play a crucial role, as families with lower income levels may face greater challenges in providing healthy food options and opportunities for physical activity, disproportionately impacting younger children^(33)^. These findings highlight the importance of tailoring public health interventions to address the unique needs of each age group, with a particular emphasis on early prevention efforts targeting primary school-aged children to mitigate the early onset and progression of obesity.

Besides, the school environment is recognised as playing a pivotal role in preventing and controlling childhood obesity, serving as a privileged space for promoting healthy habits and raising health awareness^(35)^. Educational programmes focused on nutrition and dietary practices, combined with regular physical activity, have been identified as effective strategies in addressing overweight and obesity among children and adolescents^(36)^. However, the lack of mandatory physical education in certain stages of elementary education, as outlined in Article 31 of Resolution No. 7/2010 by the Brazilian National Council of Education (CNE/CEB, 2010)^(37)^, has been highlighted as a significant challenge to implementing these initiatives. The physical education teacher has been described as a central figure in this process, bearing the responsibility of raising students’ awareness of the benefits of physical activity and identifying risks associated with excessive weight at an early stage^(38)^. Additionally, the Brazilian National Common Curricular Base (BNCC) underscores the school’s role in fostering citizenship, emphasising the importance of encouraging students to care for their bodies and understand health as both a right and a social responsibility^(39)^. The growing prevalence of childhood obesity has been noted as necessitating integrated public policies that enhance access to healthy eating, promote physical activity and strengthen the role of physical education teachers in public schools, particularly in regions such as Western Amazonia, where social and economic challenges are pronounced.

It is worth mentioning that the state public school setting of our study is served universally by the Brazilian National School Feeding Program (*Programa Nacional de Alimentação Escolar* – PNAE)^(40,41)^, a structural intervention that likely shapes children’s dietary exposures and, consequently, population-level nutritional outcomes. In Rondônia, the PNAE attains near-complete coverage across municipal and state schools and operates alongside a complementary state programme (Rondônia State School Feeding Program – PEALE-RO) that prioritises procurement from family farmers and the inclusion of regional foods and locally sourced items in school menus^(42)^. Recent national guidance and targets to curb ultra-processed foods in school meals further align the school food environment with healthier dietary patterns, suggesting that shifts in PNAE implementation may have moderated, amplified or otherwise influenced temporal trends in childhood overweight and obesity observed in this study^(40,41,43)^. Although our analysis does not permit causal attribution to PNAE activities, we therefore highlight the programme as a plausible contextual moderator of diet-related exposures in Rondônia and call for dedicated, mixed-methods evaluations of PNAE implementation, menu composition and child dietary intake to clarify its role in shaping obesity trajectories in the Amazonian context.

Through a public health relevance lens, the observed increases in obesity among girls and younger children in Rondônia have clear public health implications and call for an integrated, context-sensitive response centred on schools and primary care. Priority actions should include mandatory, high-quality physical education and daily activity opportunities in primary schools; strict enforcement of school nutrition standards with municipal procurement favouring locally sourced, minimally processed foods; gender-sensitive strategies to increase girls’ participation in physical activity (e.g. safe spaces, female-led programmes); and targeted improvements to local infrastructure that enable active recreation in peri-urban and riverine neighbourhoods. These programmatic steps must be coupled with restored, standardised school-based surveillance (annual BMI and behaviour measures), routine integration of obesity prevention into Brazilian Unified Health System (SUS) primary care visits and robust evaluation (cluster or stepped-wedge designs) to guide scale-up. Together, these measures offer a pragmatic, equity-focused pathway to reduce early-life obesity and its long-term health consequences.

Some limitations should be considered when interpreting the findings of this study. First, the annual physical examination dataset used in the analysis lacked key socio-economic and lifestyle variables, such as family income, parental educational attainment, physical activity levels and dietary habits, all of which are critical determinants of childhood overweight and obesity. While the study provides the most up-to-date prevalence estimates and highlights increasing trends in childhood overweight and obesity in Porto Velho, Rondônia, Brazil, future research should aim to incorporate detailed demographic, lifestyle and risk factor data to better understand the drivers of these trends and identify the most significant contributors to the rising prevalence. Second, this study did not include adolescents who were not attending school, a group that is generally more socio-economically disadvantaged and, consequently, at a higher risk for overweight and obesity. Including this population could have resulted in even higher prevalence rates, with disproportionate impacts observed among economically disadvantaged girls. Third, although a considerable sample size was collected in the study, the samples were not representative of adolescents in Porto Velho, Rondônia, Brazil. Fourth, because the SEDUC annual monitoring was suspended in 2020–2021, these calendar years are missing from the dataset and were excluded from the analyses. Temporal trend analyses were therefore performed using the available sequence of years (2006–2019, 2022–2024). A consequence of pandemic-related interruption may have affected trend detection and interpretability and thus represents a limitation of the present study.

Furthermore, it is important to note that the anthropometric data used in this study are directly comparable across all years analysed. The data collection process adhered to standardised protocols, with trained personnel employing calibrated equipment to ensure measurement accuracy. Additionally, unlike previous national studies, which often assess shorter time frames, this investigation spanned a more extensive period, evaluating trends over sixteen time points. This comprehensive approach strengthens the reliability of the findings and provides a more detailed understanding of the temporal patterns of excess weight and abdominal obesity.

## Conclusion

In conclusion, this study identified a significant temporal trend of increasing obesity rates among girls and children aged 6–10 years in a sample of schoolchildren from Porto Velho, the largest geographic capital in Brazil. Although no significant temporal trends were observed for overweight rates, the rising prevalence remains concerning, as early changes in nutritional status may progress to obesity over time. These findings underscore the urgent need for targeted interventions that address the unique vulnerabilities of specific age and gender groups to mitigate the long-term health impacts of excess weight in this population.

Addressing childhood obesity requires comprehensive strategies that go beyond individual lifestyle modifications, such as increasing physical activity and improving dietary habits. Interventions must also tackle the broader socio-economic, political and cultural determinants of obesity. Policies like clear nutritional labelling and reducing fat percentages in foods are steps in the right direction but need to be complemented by stronger measures, such as regulating food advertising, taxing unhealthy food products and banning the sale of energy-dense, nutrient-poor foods in schools. A truly interdisciplinary approach, incorporating sectors beyond health, is essential to effectively combat childhood obesity and reduce its lifelong repercussions.

## Data Availability

Access to the data can be made available upon request by the authors.
